# Exploring the Mechanism of Fufang Danshen Tablet against Atherosclerosis by Network Pharmacology and Experimental Validation

**DOI:** 10.3390/ph17050643

**Published:** 2024-05-16

**Authors:** Yuling Liu, Weiwei Su, Peibo Li, Xuan Zeng, Yuying Zheng, Yonggang Wang, Wei Peng, Hao Wu

**Affiliations:** Guangdong Engineering & Technology Research Center for Quality and Efficacy Reevaluation of Post-Market Traditional Chinese Medicine, Guangdong Provincial Key Laboratory of Plant Resources, School of Life Sciences, Sun Yat-sen University, Guangzhou 510275, China; liuyling26@mail2.sysu.edu.cn (Y.L.); lsssww@126.com (W.S.); lipeibo@mail.sysu.edu.cn (P.L.); zengx6@mail2.sysu.edu.cn (X.Z.); vicky_0224@126.com (Y.Z.); wangyg@mail.sysu.edu.cn (Y.W.); pweiyu929@126.com (W.P.)

**Keywords:** Fufang Danshen Tablet, atherosclerosis, network pharmacology, transcriptome, monocyte, ApoE^−/−^ mice

## Abstract

Atherosclerosis is the main pathological basis of cardiovascular diseases (CVDs). Fufang Danshen Tablet (FDT) is a traditional Chinese medicine that has been clinically used to treat CVDs for more than 40 years. Nevertheless, owing to the complexity of the ingredients, the pharmacological mechanism of FDT in the treatment of CVDs has not been fully elucidated. In this study, an integrated strategy of UFLC-Q-TOF-MS/MS, network pharmacology, molecular biology, and transcriptomics was used to elucidate the mechanisms of action of FDT in the treatment of atherosclerosis. In total, 22 absorbed constituents were identified in rat serum after oral administration of FDT. In silico, network pharmacology studies have shown that FDT regulates four key biological functional modules for the treatment of atherosclerosis: oxidative stress, cell apoptosis, energy metabolism, and immune/inflammation. In animal experiments, FDT exerted protective effects against atherosclerosis by reducing the plaque area and lipid levels in ApoE^−/−^ mice. Furthermore, we found that FDT inhibited inflammatory macrophage accumulation by regulating the expression of *Selp* and *Ccl2*, which are both involved in monocyte adhesion and migration. The inhibition of monocyte recruitment by FDT is a new perspective to elucidate the anti-atherosclerotic mechanism of FDT, which has not been adopted in previous studies on FDT. Our results may help to elucidate the therapeutic mechanism of FDT against CVDs and provide potential therapeutic targets.

## 1. Introduction

Atherosclerosis is a chronic inflammatory disease that is the main cause of cardiovascular diseases (CVDs). Over the last few years, atherosclerosis-related cardiovascular disease has emerged as a prominent cause of mortality worldwide [1]. Monocytes and their descendant macrophages play a critical role in plaque initiation at the early stage of atherosclerosis [2]. Monocytes in circulation are recruited to the vascular intima, where they differentiate into macrophages. Subsequently, macrophages excessively ingest normal and modified low-density lipoproteins (LDLs) and transform into lipid-enriched “foam cells”. The formation of foam cells is the hallmark of atherosclerotic plaques [3]. Foam cell accumulation and apoptosis eventually result in the formation of a necrotic core [4]. Therefore, targeting monocyte recruitment is becoming an effective method for the prevention and treatment of CVDs. However, monocyte recruitment is a sophisticated pathophysiological process that requires the complex regulation of multiple chemokines. Traditional Chinese medicine (TCM) has unique advantages in the treatment of cardiovascular diseases because of the synergistic effect of various bioactive components at a systemic level [5]. Furthermore, an increasing number of pharmacological studies have demonstrated the efficacy of TCM in the prevention and treatment of cardiovascular diseases [6,7,8].

Fufang Danshen Tablet (FDT), a classical TCM formula, has been used clinically to treat CVDs for more than 40 years. FDT is composed of three traditional Chinese medicines—Dan-Shen (*Salvia miltiorrhiza* Bunge), San-Qi (*Panax notoginseng* (Burk.) F.H. Chen), and Bing-Pian (Borneolum syntheticum)—in a weight ratio of 450:141:8 [9]. The herbal information is listed in Table S1. The clinical effectiveness of Dan-Shen in the treatment of CVDs has been widely recognized. A statistical study has shown that the addition of Dan-Shen preparation to standard care for patients with acute myocardial infarction resulted in a nearly 50% reduction in mortality compared to standard care alone [10]. In a study characterized by randomization, double-blinding, and placebo control, in which 110 patients with uncontrolled hypertension were included, a Fufang Danshen extract capsule (1 g twice a day) was found to aid in the treatment of hypertension by reducing systolic blood pressure and pulse rate [11]. Furthermore, some pharmacological studies have been performed to explore the mechanism of FDT. In an in vitro experiment, FDT demonstrated a significant inhibitory effect on platelet aggregation in a dose-dependent manner [9]. In a mouse model of Alzheimer’s disease induced by Aβ_25-35_, the oral administration of FDT (0.81 g/kg, 3 weeks) was found to significantly reduce neuroinflammation by inhibiting the TNF-α and IL-6 levels [12]. Salvianolic acid B, a major bioactive constituent of Dan-Shen, mitigated myocardial infarction size by upregulating B cell lymphoma 2 (Bcl-2) and upregulating Bcl-2-associated X (Bax), caspase 3, c-Jun N-terminal kinase (JNK), and p38 expression in a mouse model of myocardial ischemia/reperfusion injury [13]. However, there is much to uncover regarding the mechanism of action, as the various chemical constituents of the multicomponent FDT exhibit diversity in their corresponding treatment targets.

**Figure 1:**
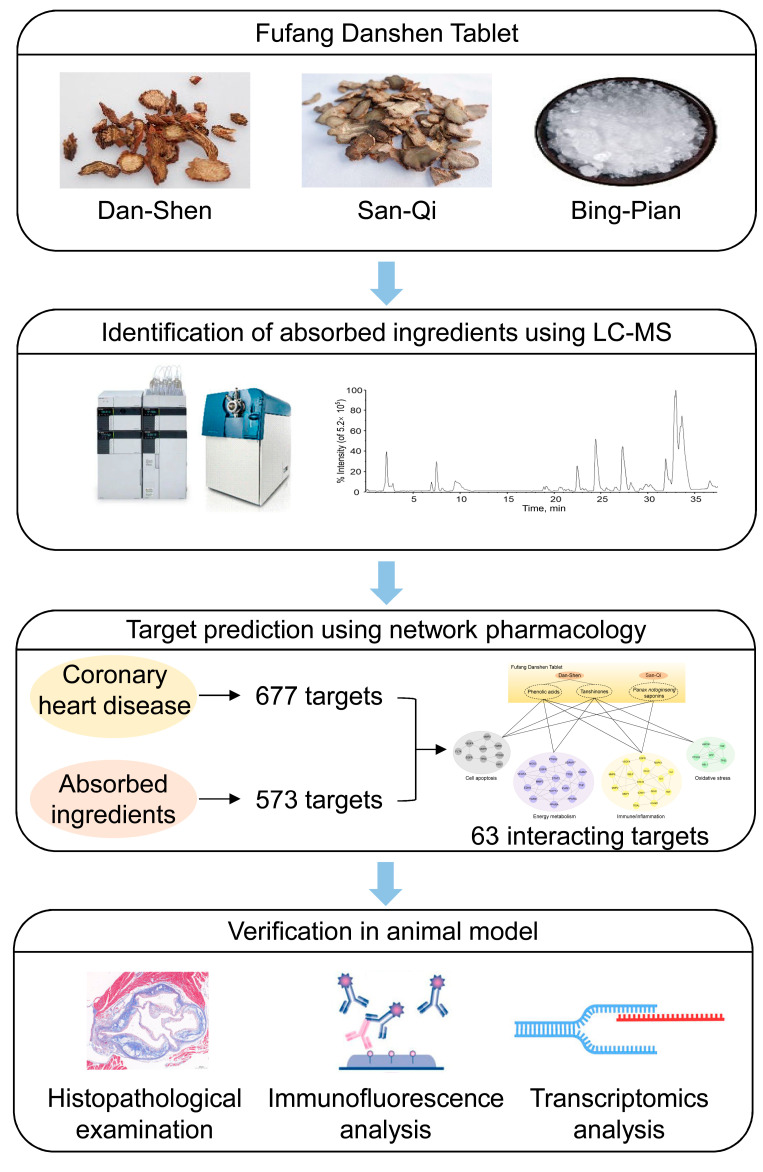
Work scheme for exploring the pharmacological mechanism of Fufang Danshen Tablet against atherosclerosis.

In order to investigate the anti-atherosclerotic effects of FDT and elucidate the underlying mechanism, a systematic approach was employed by integrating network pharmacology and experimental validation. The absorbed constituents of FDT in rat serum were identified using ultra-fast liquid chromatography/quadrupole-time-of-flight tandem mass spectrometry (UFLC-Q-TOF-MS/MS). Network pharmacology was performed to screen the potential targets of the absorbed constituents and obtain therapeutic candidates for FDT against atherosclerosis. Apolipoprotein E (ApoE)-deficient mice fed a high-fat diet (HFD) were selected to establish an atherosclerosis model. Biological, histological, immunofluorescence, and transcriptomic approaches have been used to validate the pharmacological effects and molecular mechanisms of FDT in atherosclerosis. This study will contribute to the comprehension of the therapeutic mechanisms of FDT against CVDs and identify potential therapeutic targets for CVDs. A detailed workflow is shown in Figure 1.

## 2. Results

### 2.1. Identification of the Absorbed Constituents in Rat Serum

**Figure 2:**
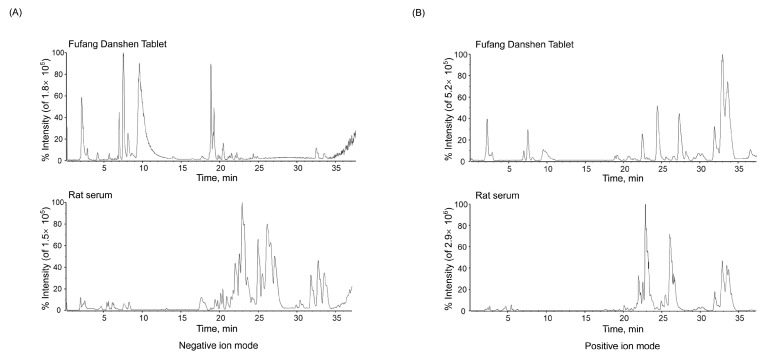
Base peak chromatogram (BPC) of Fufang Danshen Tablet (FDT) and rat serum after oral administration of FDT. (**A**) The negative ion mode. (**B**) The positive ion mode.

To determine the composition of FDT, three batches of FDT samples were analyzed using UFLC-Q-TOF-MS/MS in both positive and negative ionization modes (Figure 2). A total of 81 ingredients were unambiguously or tentatively identified (Table S2). Phenolic acids, tanshinones, and *Panax notoginseng* saponins were the major chemical ingredients of FDT. All compounds could be detected in the individual constituent herbs. According to the composition results, 22 absorbed constituents were identified in rat serum after oral administration of FDT. The absorbed components included phenolic acids, tanshinones, and *Panax notoginseng* saponins. Detailed information is provided in Table S3.

### 2.2. Network Pharmacology Results

#### 2.2.1. Target Acquisition of Absorbed Constituents Related to Atherosclerosis

For drugs, release into circulation is a prerequisite for their effectiveness. Hence, the absorbed constituents were selected for network pharmacology investigation. A total of 573 targets of the 22 absorbed constituents were extracted from the TCMSP and SwissTargetPrediction databases (Table S4). After selecting and eliminating redundancy, a total of 670 targets associated with atherosclerosis were obtained from the DrugBank, GeneCards, and OMIM databases (Table S5). When the drug targets intersected with the disease targets, 63 overlapping targets were identified (Table S6).

#### 2.2.2. FDT Regulates the Biological Functional Modules of Immune/Inflammation, Oxidative Stress, Cell Apoptosis, and Energy Metabolism in the Treatment of Atherosclerosis

To reveal the pharmacological effects of FDT on atherosclerosis, GO and KEGG pathway enrichment analyses were performed with 63 interacting targets using the David database (Tables S7 and S8). The absorbed constituent–target–pathway network was constructed (Figure S1). As shown in Table 1, the KEGG signaling pathways and GO biological processes were significantly enriched, with a primary focus on oxidative stress, cell apoptosis, energy metabolism, and immune/inflammation. Therefore, the targets were classified into four biological functional modules. Of these, the overproduction of reactive oxygen species results in oxidative stress, thereby promoting atherosclerosis through the oxidation of lipids and DNA, the impairment of endothelial function, and the induction of inflammation [14]. The apoptosis of vascular endothelial cells, macrophages, or vascular smooth muscle cells is a key feature of the progression of atherosclerotic plaques, contributing to the formation of necrotic core [15]. The energy metabolism disorder of endothelial cells promotes neovascularization and activates hypoxia-inducible factors that increase atherosclerotic lesion size [16]. Immune response and inflammation are key components in the pathogenesis of atherosclerosis. In the early phase of plaque formation, monocytes adhere and migrate to the damaged endothelium to form macrophage foam cells, which play a crucial step in triggering atherosclerosis [17]. Therefore, targeting monocyte adhesion and migration is considered a promising strategy to treat atherosclerosis.

**Figure 3:**
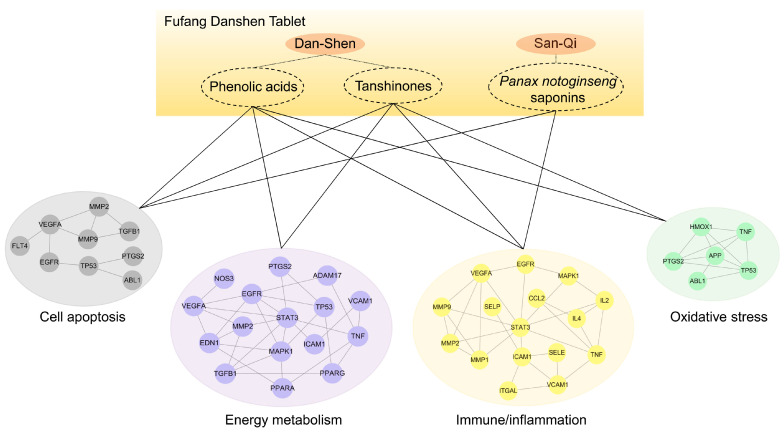
The absorbed constituents–biological functional module–molecule network. The network implies the mechanism of FDT in the treatment of atherosclerosis. The genes in each biological functional module were analyzed using the protein–protein interaction network.

For a more visual understanding, an absorbed constituent–biological functional module network was constructed, as shown in Figure 3. These results could help us to understand the pharmacological effects of FDT from a holistic point of view. Dan-Shen regulates immune/inflammation, oxidative stress, cell apoptosis, and energy metabolism modules. San-Qi mediates cell apoptosis and immune/inflammation modules. Notably, some studies have confirmed the network pharmacological predictions. For instance, Dan-Shen injection exerts anti-inflammatory effects by decreasing the expression of MMP2, MMP9, and myeloperoxidase (MPO) in a rat model of post-myocardial infarction [18]. *Panax notoginseng* saponins (PNSs) can inhibit inflammation by suppressing the HIF-1α/PKM2/STAT3 signaling pathway in photothrombotic stroke mice [19]. These studies indicate that FDT has a material basis for regulating immune/inflammation modules, warranting further investigation.

### 2.3. In Vivo Experimental Validation

#### 2.3.1. FDT Lowers the Serum Lipid Levels in ApoE^−/−^ Mice

**Figure 4:**
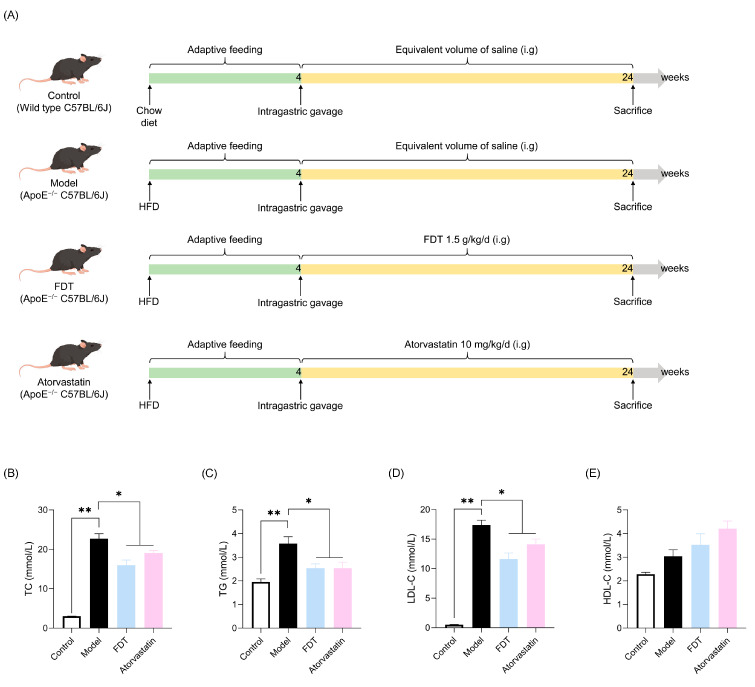
FDT reduces lipid levels in ApoE^−/−^ mice. (**A**) Flow chart of the animal experiment. HFD: high-fat diet. (**B**) TC in serum. (**C**) TG in serum. (**D**) LDL-C in serum. (**E**) HDL-C in serum. All data are expressed as means ± SEM, * *p* < 0.05, ** *p* < 0.01. TC: total cholesterol, TG: triacylglycerol, LDL-C: low-density lipoprotein cholesterol, HDL-C: high-density lipoprotein cholesterol.

To confirm the effect of FDT on atherosclerosis progression, animal experiments were conducted as described in Figure 4A. ApoE^−/−^ mice were given a high-fat diet for 20 weeks with or without drug intervention. The serum lipid levels, including TC, TG, LDL-C, and HDL-C, were examined in each group, as shown in Figure 4B–E. In comparison to the control group, the model group exhibited a significant increase in serum levels of total cholesterol (TC), triglycerides (TG), and low-density lipoprotein cholesterol (LDL-C) (*p* < 0.01), while high-density lipoprotein cholesterol (HDL-C) levels showed no significant difference. Conversely, the FDT group demonstrated a significant decrease in serum TC, TG, and LDL-C levels compared to the model group (*p* < 0.05).

#### 2.3.2. FDT Reduces Atherosclerotic Plaque Development in ApoE^−/−^ Mice

**Figure 5:**
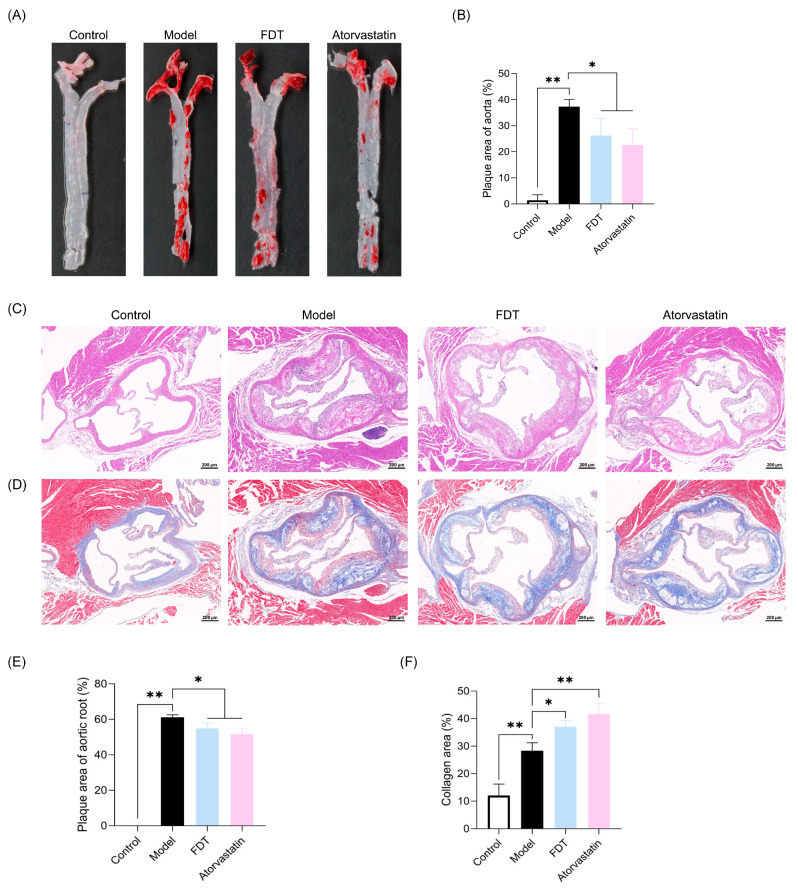
FDT attenuates atherosclerotic plaques in ApoE^−/−^ mice. (**A**) Representative images of Oil red O staining of thoracic aortas. (**B**) Quantification of the plaque area of thoracic aortas. (**C**) Representative images of aortic root sections stained with hematoxylin and eosin (H&E), scale bar = 200 μm. (**D**) Representative images of aortic root sections stained with Masson, scale bar = 200 μm. (**E**) Quantification of the plaque area of aortic roots using H&E staining. (**F**) Quantification of collagen fibers areas in the plaques using Masson Trichromic staining. All data are expressed as means ± SEM. * *p* < 0.05, ** *p* < 0.01.

Atherosclerotic lesions in the thoracic aorta and aortic root were examined after 20 weeks of administration. Figure 5A,B show that a high-fat diet induced severe thoracic aortic lesions in ApoE^−/−^ mice (*p* < 0.01). The administration of FDT to ApoE^−/−^ mice resulted in a notable reduction in atherosclerotic lesion development in the thoracic aorta (*p* < 0.05). Similar findings were obtained in the HE staining of the aortic roots. The results depicted in Figure 5C,E indicate that the proportion of the lesion area in ApoE^−/−^ mice treated with FDT was significantly reduced compared to the model group (*p* < 0.05). Masson staining of the aortic roots was performed, and the results showed that FDT treatment significantly increased the proportion of collagen fibers in plaques at the aortic root (*p* < 0.05), indicating enhanced plaque stability (Figure 5D,F).

#### 2.3.3. FDT Reduces the Number of CD68-Positive Macrophages in the Plaque

**Figure 6:**
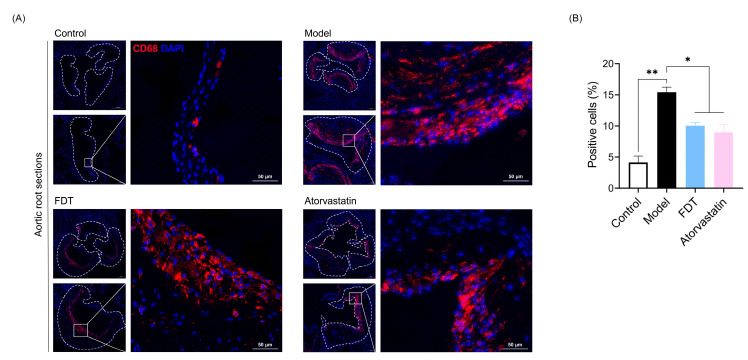
CD68 is downregulated by FDT in arteriosclerosis model ApoE^−/−^ mice. (**A**) Aortic root sections from mice are stained with CD68 (macrophages, red). The nuclei are stained with DAPI (blue). Scale bars are indicated as in the figure. (**B**) The number of CD68-positive cells was counted and analyzed, and data are expressed as mean ± SEM, * *p* < 0.05, ** *p* < 0.01.

Recruitment of circulating monocytes into the vessel intima is a critical process that contributes to macrophage accumulation in atherosclerotic plaques. Immunofluorescence staining for CD68 was performed to assess macrophage infiltration in the aortic root. As shown in Figure 6, compared to the control group, the number of CD68-positive cells was dramatically increased in the model group (*p* < 0.01). FDT treatment significantly decreased the number of CD68-positive cells (*p* < 0.05). The inhibitory effect of FDT was comparable to that of atorvastatin. Our results indicate that FDT could inhibit the accumulation of inflammatory macrophages.

#### 2.3.4. Transcriptome Analysis Verified the Mechanisms of FDT in ApoE^−/−^ Mice with High-Fat Diet Feeding

**Figure 7:**
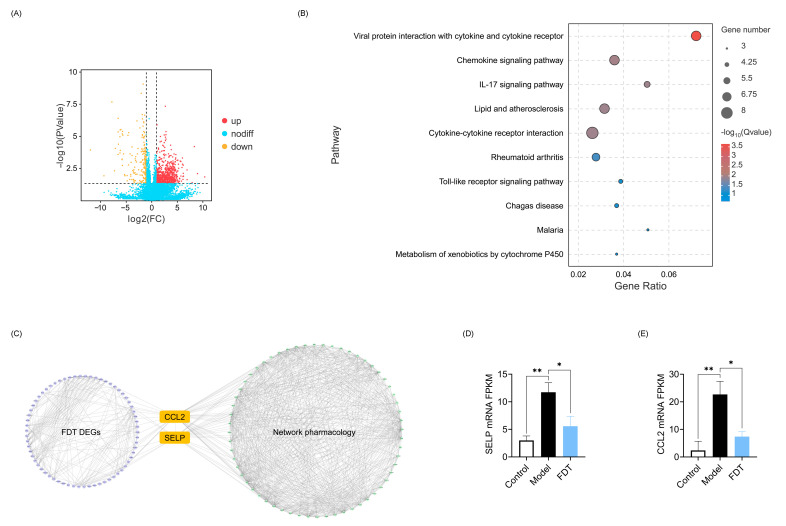
Transcriptomics analysis of FDT on atherosclerosis. (**A**) Volcano plot of differential expression genes between model and FDT groups. There are 33 upregulated genes and 66 downregulated genes. (**B**) KEGG pathway enrichment of differential expression genes between Model and FDT group. (**C**) Joint analysis of the differential expression genes (Model vs. FDT) and potential targets from network pharmacology. The purple circles represent the DEGs (Model vs. FDT) from transcriptomics. SELP and CCL2 were verified by transcriptomics. (**D**) SELP and (**E**) CCL2 mRNA level in ApoE^−/−^ mice. Data are expressed as mean ± SEM, * *p* < 0.05, ** *p* < 0.01.

To confirm the potential mechanisms of FDT against atherosclerosis obtained via network pharmacology, transcriptome analysis was conducted using an atherosclerotic model established in ApoE^−/−^ mice. A total of 3193 differentially expressed genes (DEGs) were identified in the comparison between the control and model groups, comprising 2088 upregulated genes and 1105 downregulated genes. Meanwhile, a total of 99 DEGs were identified by comparing the model and FDT-treated groups, of which 33 were upregulated and 66 were downregulated (Figure 7A). Detailed information is provided in Table S9. KEGG enrichment analysis was conducted on the DEGs, and 24 pathways were obtained at *p* < 0.05. Most DEGs were primarily enriched in pathways related to inflammation and immune response, such as the viral protein interaction with cytokines and their receptor pathway, chemokine signaling pathway, IL-17 signaling pathway, and cytokine–cytokine receptor interaction pathway. The top ten enriched KEGG pathways of DEGs in the model vs. FDT are shown in Figure 7B. These results further support the findings of network pharmacology that FDT can regulate the biological functional modules of immune/inflammation in the treatment of atherosclerosis.

Two targets, P-selectin (SELP) and C-C motif chemokine 2 (CCL2), obtained from network pharmacology were verified through transcriptome sequencing (Figure 7C). The gene expression ratios of *Selp* and *Ccl2* are exhibited in Figure 7D,E. Compared to the control group, FPKM values of *Selp* and *Ccl2* were significantly increased in the model group (*p* < 0.01). FDT treatment significantly decreased the expression levels of *Selp* and *Ccl2*. SELP and CCL2 are involved in the atherosclerotic inflammatory process [20]. SELP, an adhesion receptor usually expressed on the activated endothelium, is crucial for facilitating the firm adhesion of monocytes to the luminal surface of the endothelium [21]. CCL2, synthesized by endothelial cells, smooth muscle cells, and macrophages within atherosclerotic lesions, interacts with its receptor CCR2 on circulating monocytes, facilitating their migration across the vascular endothelium and into the plaques [22]. The results suggest that FDT may treat atherosclerosis by inhibiting monocyte adhesion and migration.

#### 2.3.5. Molecular Docking Simulations

**Figure 8:**
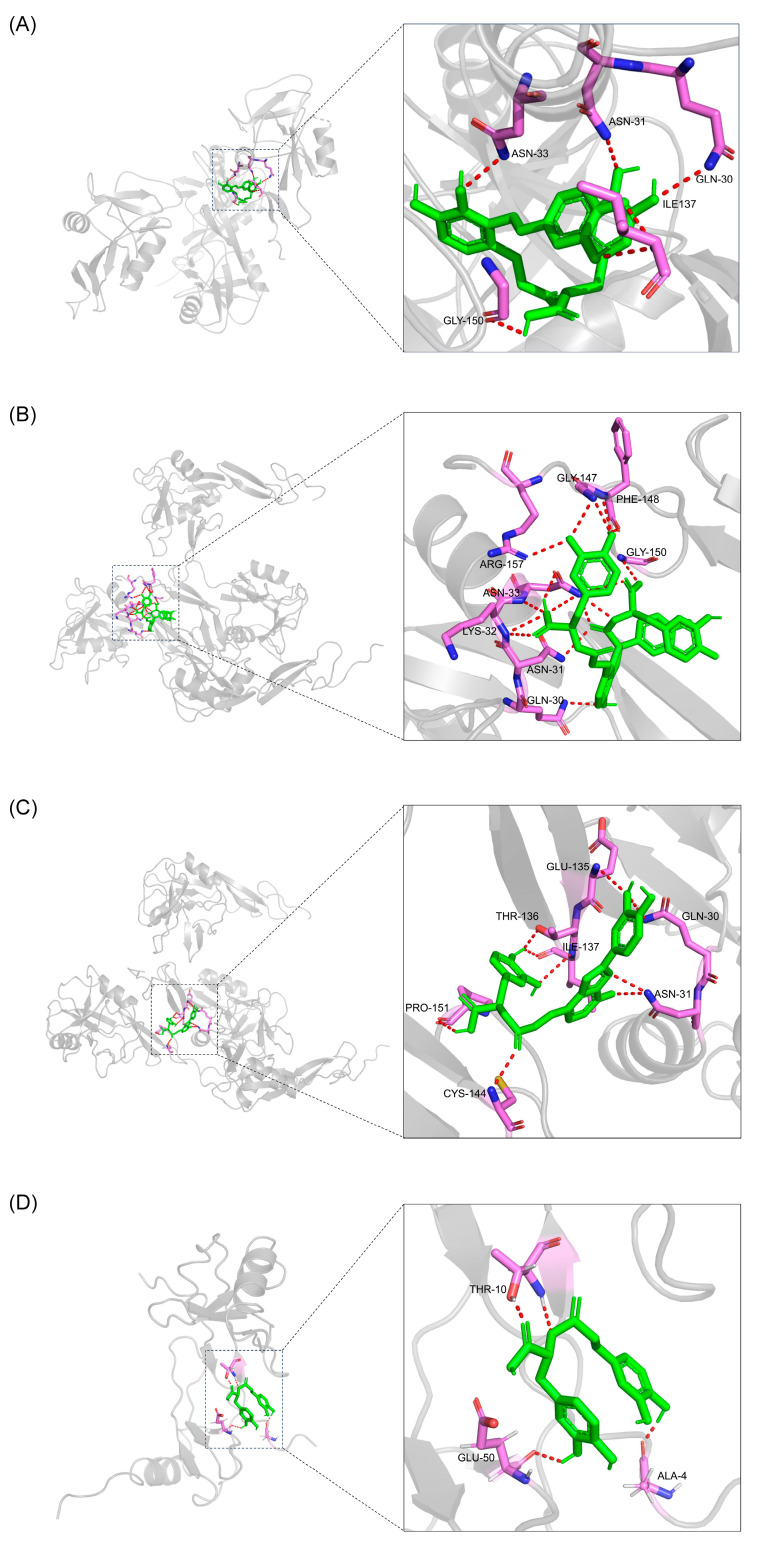
Molecular docking results of absorbed constituents with SELP and CCL2. (**A**) Salvianolic acid A and SELP. (**B**) Salvianolic acid B and SELP. (**C**) Salvianolic acid C and SELP. (**D**) Rosmarinic acid and CCL2.

SELP and CCL2 obtained from network pharmacology were verified using transcriptome analysis. Therefore, SELP and CCL2 were selected as the active targets for FDT against atherosclerosis. According to the results of the network pharmacology analysis, salvianolic acids A, B, and C interacted with SELP, while rosmarinic acid interacted with CCL2. These four absorbed constituents were selected as ligands for molecular docking with SELP and CCL2. The results were expressed as the Vina score, with lower scores representing higher binding affinities. Table 2 shows the docking scores, and Figure 8 displays the interaction and specific amino-acid-binding sites in optimal docking after visualization. The results showed that salvianolic acids A, B, and C interacted with SELP, with docking scores of −8.1, −8.2, and −8.7, respectively, indicating that salvianolic acids A, B, and C strongly interacted with SELP. Docking analysis of rosmarinic acid and CCL2 also showed high docking activity, with a score of −6.5. Together, these findings suggest that the four compounds were pharmacodynamic constituents of FDT that act on SELP and CCL2.

## 3. Discussion

This study integrated UFLC-Q-TOF-MS/MS, network pharmacology, and experimental validation to investigate the key active ingredients of FDT and their potential targets in atherosclerosis. A remarkable finding was that FDT exerted anti-atherosclerotic effects by inhibiting monocyte adhesion and migration. The inhibition of monocyte recruitment by FDT is a new perspective to elucidate the anti-atherosclerotic mechanism of FDT, which has not been adopted in previous studies on FDT. Our results may contribute to the comprehension of the therapeutic mechanisms of FDT against CVDs and aid in the identification of potential therapeutic targets for CVDs.

Comprehensive investigations of absorption, distribution, metabolism, and excretion (ADME) in vivo are of great value for evaluating the efficacy and safety of drugs. In traditional Chinese medicine, the active ingredients generally need to be absorbed and reach the target tissue at a workable concentration to exert therapeutic effects [23]. Therefore, it is imperative to identify and elucidate the constituents with substantial exposure to blood or tissue after administration in pharmacological studies of TCM. In this study, most of the phenolic acids and tanshinones identified in FDT samples could be found in rat serum. However, nineteen *Panax notoginseng* saponins were identified in FDT samples, only four of which were found in rat serum. It is indicated that the oral absorption of saponins was extremely low. Song et al. [24] detected three saponins in dog plasma after oral administration of FDT at a clinical equivalent dose. The C_max_ values for notoginsenoside R_1_ and ginsenoside Rg_1_ and Rb_1_ were 1.91, 3.34, and 28.6 ng/mL, respectively, which were in the low nanogram per milliliter levels. This result aligned with our study. In another study, Guo et al. [25] quantitative analyzed the metabolites of *Panax notoginseng* saponins in rat plasma after oral administration with *Panax notoginseng* saponin extract at a dose of 1.535 g/kg. The results showed that four metabolites, including ginsenoside F_1_, ginsenoside Rh_2_, ginsenoside compound K, and protopanaxatriol, could be detected in normal rat plasma at 12 h after drug administration, but not in pseudo germ-free rat plasma. It is indicated that *Panax notoginseng* saponins can be biotransformed by the gut microbiota. However, in our study, the metabolites of *Panax notoginseng* saponins were not found in rat serum after oral administration of FDT. This is likely due to the different doses and the sampling time points.

Network pharmacology is considered as an effective and integrated approach for investigating interactions among drugs, targets, and diseases based on systems biology. It is suitable for exploring the complicated mechanisms of TCM formulas for treating complex diseases [26]. The network pharmacology study showed that FDT mainly intervenes in the development of atherosclerosis by regulating immune/inflammation, oxidative stress, energy metabolism, and cell apoptosis. In terms of immune/inflammation, we found that 13 absorbed constituents including salvianolic acids A, B, and C; rosaminic acid; tanshinone II_A_; and cryptotanshinone could inhibit the adhesion and migration of monocytes in atherosclerosis by regulating adhesion molecules such as ICAM1 and VCAM1. Yang et al. [27] found that tanshinone II_A_ can inhibit the expression of ICAM1 and VCAM1 in TNF-α-induced endothelial progenitor cells, thereby inhibiting the adhesion of monocytes to endothelial progenitor cells. In human umbilical vein endothelial cells induced by TNF-α, it has been found that salvianolic acid B could significantly reduce the mRNA levels of ICAM1 and P-NF-κB p65, indicating that salvianolic acid B inhibits the NF-κB/NLRP3 signaling pathway and reduces the inflammatory response [28]. In terms of oxidative stress, the network pharmacology study showed that 14 absorbed constituents, including salvianolic acids A, B, and C; tanshinone I; tanshinone II_A_; and rosmarinic acid, could regulate PTGS2, APP, ABL1, HMOX1, TP53 and other targets of oxidative stress. Oxidative stress can increase the expression of PTGS2, leading to the promotion of prostaglandin synthesis [29]. It was found that salvianolic acid injection could decrease the level of prostaglandin E2 by inhibiting PTGS2 activity in lipopolysaccharide-induced RAW264.7 macrophages [30]. In terms of energy metabolism, the network pharmacology study showed that 17 absorbed constituents including salvianolic acids A, B, and C and tanshinone II_A_ can act on the HIF-1 signaling pathway. In endothelial cells, tanshinone II_A_ increased the mRNA level of GLUT-1, which promoted the activation of the HIF-1α signaling pathway, leading to enhanced glucose uptake [31]. In terms of cell apoptosis, ischemia, hypoxia, inflammation, and oxidative stress caused by atherosclerosis can all induce apoptosis, while excessive apoptosis further aggravates atherosclerosis [15]. The network pharmacology study showed that 20 absorbed constituents including tanshinone II_A_, salvianolic acid, and ginsenoside Rb_1_ can regulate apoptosis-related targets such as TP53, MMP9, PPARG, and FLT4. TP53 is a tumor suppressor gene that encodes the p53 protein. In hypertrophic cardiomyopathy, the expression of p53 is significantly increased, which induces apoptosis of vascular endothelial cells and myocardial fibrosis [32]. Liu et al. [33] studied the protective effect of tanshinone II_A_ on hypoxia–reoxygenation injury in H9c2 cells. The results showed that tanshinone II_A_ could inhibit cell apoptosis by reducing the mRNA levels of TP53, Akt1, and c-Jun. The literature discussed above preliminarily proves the reliability of network pharmacology. Therefore, it is proposed that FDT may have synergistic effects in the treatment of atherosclerosis.

ApoE^−/−^ mice have been extensively used for atherosclerosis studies because they spontaneously develop atherosclerotic lesions with characteristics similar to humans [34]. Therefore, ApoE^−/−^ mice fed a HFD were selected to study the anti-atherosclerosis mechanism of FDT. The results showed that FDT suppressed the gene expression of SELP and CCL2, suggesting that the reduced recruitment of monocytes during atherosclerotic plaque formation may be the underlying mechanism by which FDT exerts its anti-atherosclerotic effects. Molecular docking was performed to investigate the binding affinity of the absorbed constituents to SELP and CCL2. Salvianolic acids A, B, and C demonstrated significant binding affinities with SELP, while rosmarinic acid exhibited strong binding affinities with CCL2. The results suggest that these four absorbed constituents may have interacted with SELP and CCL2. Several lines of evidence support our speculation. Rosmarinic acid could suppress the expression of CCL2 in bone-marrow-derived dendritic cells (BMDCs) induced by lipopolysaccharides [35]. Salvianolic acid B attenuated SELP and other biomarkers to decrease inflammation in endothelial cells triggered by activated platelets [36]. In a clinical trial study, salvianolic acid A inhibited the plasma level of SELP in patients with type 2 diabetes mellitus [37]. In a mouse model of myocardial ischemia/reperfusion injury, salvianolic acid A exerted an anti-inflammatory effect by reducing serum levels of SELP and other cytokines [38]. Taken together, these results suggest that FDT may inhibit atherosclerosis by targeting SELP and CCL2.

Targeting SELP and CCL2 may be a potential intervention for atherosclerosis therapy. Monocytes play an essential role in plaque formation and are regulated by a range of endothelial adhesion molecules and chemokines [2]. Ample evidence from epidemiological, preclinical, and clinical studies indicates that some selectins and chemokines are associated with a risk of cardiovascular disease and mediate the pathological process of atherogenesis [39]. For example, P-selectin is a vascular cell adhesion molecule that regulates monocyte adhesion to the endothelium. Epidemiological studies have demonstrated that elevated plasma P-selectin levels correlate with the development of myocardial infarction [40]. Preclinical evidence has shown that P-selectin-deficient mice inhibit atherosclerotic plaque formation [41]. Further clinical trials have indicated that the P-selectin inhibitor inclacumab demonstrates potential in mitigating myocardial damage after percutaneous coronary intervention, as found in a phase II trial [42]. Another chemotactic factor involved in the recruitment of monocytes, CC chemokine ligand 2 (CCL2), promotes monocyte migration across the vascular endothelium. The epidemiologic investigation uncovered that higher CCL2 levels in the blood and atherosclerotic plaques increases the risk of plaque vulnerability and cardioembolic stroke [43,44]. In preclinical studies, *Ccl2*-deficient mice showed lower lipid deposition and macrophage accumulation in atherosclerotic plaques. Conversely, the overexpression of *Ccl2* in ApoE^−/−^ mice resulted in the acceleration of atherosclerosis [22]. Similar results were obtained with other selectin and chemokines such as, E-selectin, CCR2, CCL5, etc. [45,46]. Therefore, targeting monocyte recruitment is an effective strategy in the management of cardiovascular disease.

This study has some limitations. Firstly, the interactive effect of the absorbed constituents can only partially represent the actions of FDT. The interaction between other components, the contribution of metabolites, and drug-induced changes in blood proteins and lipids are also important mediators, which require further investigation. Secondly, Bing-Pian, the adjuvant ingredient in FDT, did not acquire the target profile because we did not identify any constituent in rat serum with UFLC-Q-TOF-MS/MS. The main reasons for this include the low dosage (1.3% in weight) of Bing-Pian used in the prescription and the volatility of borneol. Nevertheless, the pharmacology of Bing-Pian cannot be ignored. It has not only been used in traditional medication for the treatment of CVDs with a broad range of pharmacological effects such as vasorelaxant, anti-inflammatory, and sedation effects [47], but also usually serves as a guide drug, which can improve absorption and influence the distribution of other ingredients in the formula [48]. For example, Bing-Pian could significantly shorten the *t*_max_ and increase the *c*_max_ of Tanshinone II_A_, salvianolic acid B, and Ginsenoside Rg_1_ in rat plasma and brains [49]. Thus, it could be seen that Bing-Pian could induce the effective ingredients to play a quicker therapeutic role and produce synergistic effects in CVDs. In subsequent research endeavors, further experiments will be undertaken to corroborate the mechanisms of action of the key compounds in FDT.

## 4. Materials and Methods

### 4.1. Identification of Absorbed Ingredients from FDT in Rat Serum

#### 4.1.1. FDT Samples

FDT samples were uncompressed granules containing herbal material and excipients, which were provided by Hutchison Whampoa Baiyunshan Chinese Medicine Co., Ltd. (Guangzhou, China). Accurately weighed FDT (0.5 g) was extracted ultrasonically with 50 mL of 70% (*v/v*) methanol–water solution for 30 min at room temperature. Following centrifugation, the supernatant underwent filtration using a 0.22 μm microporous filter. A 2 μL aliquot of the filtrate was utilized for UFLC-Q-TOF-MS/MS analysis. Three batches of FDT were analyzed.

#### 4.1.2. Reference Standards

An individual reference standard (Table S10) was weighed and dissolved in methanol to prepare a standard stock solution. All standard stock solutions were mixed at appropriate concentrations, except for the isomers. The mixture was filtered through a 0.22 μm microporous filter. A 2 μL aliquot of the filtrate was utilized for UFLC-Q-TOF-MS/MS analysis.

#### 4.1.3. Rat Serum Samples

Twelve healthy male Sprague Dawley rats (SPF grade, 200 ± 20 g) were purchased from the Laboratory Animal Center of the Sun Yat-sen University (Guangzhou, China). Animal facilities and protocols were approved by the Institutional Animal Care and Use Committee of Sun Yat-sen University (approval number: 2022-000015). All rats were kept in an environmentally controlled breeding room (22 ± 2 °C, 40–70% relative humidity, 12 h light/dark cycle) with unlimited access to food and water. After seven days of acclimation, the rats were randomly split into two groups: control and FDT. The granules of FDT were mixed with saline (0.3 g of granules with 1 mL of saline solution) and sonicated for 30 min. The suspension was fully blended again prior to use. The FDT group was intragastrically administered FDT at a dose of 2.3 g/kg/day for a duration of seven consecutive days, whereas the control group was administered an equivalent volume of saline. Following the final administration on the seventh day, blood samples were collected at 30, 90, and 180 min, respectively. Blood samples of 0.5 mL were obtained from the orbital vein at 30 and 90 min. At the time point of 180 min, rats were anesthetized via intraperitoneal injection of 1.5% pentobarbital sodium (0.3 mL/100g body weight) and blood samples were collected from the hepatic portal vein. The serum was isolated through centrifugation at 3000 rpm for 10 min and stored at −80 °C until analysis.

Serum samples collected at the same time points were combined. Acetonitrile (200 μL) was added to 100 μL of the mixed serum, vortexed for 3 min, and centrifuged at 4 °C. The supernatant was obtained and desiccated under a nitrogen atmosphere at 37 °C until fully dried. The resulting residue was dissolved in 100 μL of acetonitrile and centrifuged at 4 °C. A 10 μL aliquot of the supernatant was utilized for UFLC-Q-TOF-MS/MS analysis.

#### 4.1.4. UFLC-Q-TOF-MS/MS Analysis Conditions

The analysis was performed using a UFLC XR instrument equipped with an online degasser, binary pump, and autosampler (Shimadzu Corp., Kyoto, Japan). The samples were separated using a Kinetex C_18_ column (3.0 mm × 150 mm, 2.6 μm, 100 Å; Phenomenex, Torrance, CA, USA) with a column temperature of 30 °C. The mobile phase consisted of 0.1% aqueous formic acid (*v/v*) (A) and acetonitrile (B), and a linear gradient elution was optimized as follows: 0–1 min, 10–15% B; 1–3 min, 15–28% B; 3–15 min, 28–30% B; 15–17 min, 30–66% B; 17–27 min, 66–80% B; 27–33 min, 80–87% B; and 33–37 min, 87–100% B. The flow rate was set at 0.3 mL/min.

The UFLC-separated compounds were detected using a hybrid triple quadrupole time-of-flight mass spectrometer (Triple TOF 5600 plus, AB SCIEX, Foster City, CA, USA) equipped with an electrospray ion source (ESI). MS identification was conducted with the following settings: ion source gases 1 and 2 were both set at 55 psi, while the curtain gas was set at 35 psi. The temperature of the ion source was set at 550 °C. The capillary voltages in the positive and negative ion modes were 5500 V and −4500 V, respectively. The collision energy was 35 eV (with a spread of 25 eV). TOF-MS data were collected from *m*/*z* 100 to 1500 Da. Data acquisition and processing were conducted using Analyst TF 1.6 and PeakView 2.2 software (AB SCIEX, Foster City, CA, USA), respectively. Absorbed constituents were identified by examining the chromatographic retention time; MS/MS fragmentation pattern; relevant literature; available reference standards; and mass spectral library, such as PubChem and HMDB [50].

### 4.2. Network Pharmacology Analysis

#### 4.2.1. Acquisition of Targets and PPI

The absorbed constituents were entered into TCMSP [51] and the SwissTargetPrediction [52] database to obtain target names. Meanwhile, the targets of atherosclerosis were collected from Online Mendelian Inheritance in Man (OMIM) [53], Drugbank [54], and Genecards [55]. The intersection targets of absorbed constituents and disease were obtained using Venny 2.1 [56].

#### 4.2.2. Functional Enrichment

The intersection targets were uploaded to the DAVID database to execute Gene Ontology (GO) and Kyoto Encyclopedia of Genes and Genomes (KEGG) pathway enrichment analyses [57]. The specific species was restricted to Homo sapiens. The *p* values were set at <0.05. The GO enrichment analysis included the cellular component (CC), molecular function (MF), and biological processes (BPs).

#### 4.2.3. Construction of the Absorbed Constituents–Biological Functional Module Network

The construction of the network was referenced from Zhou’s study [58]. In terms of the semantic meaning, each GO term and KEGG pathway was categorized into different biological functional modules. The genes from the same biological functional module were uploaded to the STRING database (version 11.5) in order to construct the protein–protein interaction (PPI) network [59]. The organism was selected as “Homo sapiens” with a minimum interaction score of 0.4. The absorbed constituents, targets, and biological functional modules were imported into Cytoscape 3.7.2 to construct the network.

In the network, a certain type of compound was connected to the herb if it was identified within it. One type of compound was connected to one biological functional module if it was significantly enriched (*p* < 0.05) in the biological functional module. A gene was connected to another if it had an edge in the protein–protein interaction network. It should be noted that we have only shown some of the intersection genes.

### 4.3. In Vivo Experimental Validation

#### 4.3.1. Animal Model Construction and Sample Collection

Male ApoE^−/−^ and wild-type (C57BL/6J) mice aged four weeks were purchased from the Guangdong Medical Laboratory Animal Center (Guangzhou, China). Animal experiments were carried out in accordance with protocols approved by the Institutional Animal Care and Use Committee of Sun Yat-sen University. All mice were housed in a specific pathogen-free (SPF) environment as previously described. ApoE^−/−^ mice were provided with a high-fat diet containing 42% of total calories from fat (Guangdong Medical Laboratory Animal Center, Guangzhou, China). Wild-type C57BL/6 mice were given standard rodent chow and used as the control group. At eight weeks old, ApoE^−/−^ mice were randomly allocated to three groups (nine mice/group): the model group, FDT group, and atorvastatin group. FDT (Hutchison Whampoa Baiyunshan Chinese Medicine Co., Ltd., Guangzhou, China) was mixed with sterile saline (0.3 g of granules with 1 mL of saline solution) and sonicated for 30 min. The suspension was fully blended again prior to use. The atorvastatin tablet (Pfizer Pharmaceuticals LLC, Canonsburg, PA, USA) was dissolved in sterile saline and fully blended prior to use. The FDT group received intragastric administration at a dosage of 1.5 g/kg/day [60], while the atorvastatin group received intragastric administration at a dosage of 10 mg/kg/day. The control and model groups were administered an equivalent volume of 0.9% saline. All mice received intragastric administration once daily. Following a 20-week intervention period, the mice underwent a 12-h fasting period and were subsequently anesthetized via intraperitoneal injection of 1.5% pentobarbital sodium (0.5 mL/100 g body weight). Blood, aorta, and heart samples connected to the aortic root were obtained.

#### 4.3.2. Serum Lipids

Blood samples were obtained via extraction of the eyeball and centrifugation at 3000 rpm for 10 min. Serum samples were obtained for the purpose of lipid analysis, with measurements conducted for total cholesterol (TC), triglycerides (TG), low-density lipoprotein cholesterol (LDL-C), and high-density lipoprotein cholesterol (HDL-C) utilizing commercially available kits (Nanjing Jiancheng, Najing, China).

#### 4.3.3. Oil Red O Staining

Aorta samples were dissected at the proximal end of the aortic arch and bifurcation, and excess fat and tissue were removed under a microscope; the samples were then fixed with 4% paraformaldehyde. After washing with PBS, aortas were cut longitudinally along the vessel wall and subjected to staining with Oil Red O liquid for 60 min at 37 °C. Subsequently, images were acquired utilizing a camera, and Image-Pro Plus 6.0 software (Media Cybernetics, Rockville, MD, USA) was employed for the determination of the percentage of positively stained area.

#### 4.3.4. Histopathological Analysis

The heart samples connected to the aortic root underwent fixation in 4% paraformaldehyde and subsequent embedding in paraffin. Paraffin-embedded tissue blocks were cut into 4 µm sections from the middle of the left ventricle to the ascending aorta using a paraffin slicing machine (Leica RM2016, Leica Mikrosysteme Vertrieb GmbH, Wetzlar, Germany). Subsequently, hematoxylin and eosin (HE) and Masson’s trichrome staining were performed separately using an HE staining kit (G1003, Servicebio Technology Co., Ltd., Wuhan, China) and Masson’s trichrome staining kit (G1006, Servicebio Technology Co., Ltd., Wuhan, China). Images were scanned using an automatic digital slide scanner (Pannoramic MIDI, 3DHISTECH Ltd., Budapest, Hungary) to observe atherosclerotic plaques and collagen fibers. Image-Pro Plus 6.0 software (Media Cybernetics, USA) was utilized for the quantification of the proportion of positively stained area.

#### 4.3.5. Immunofluorescence Analysis

The aortic root sections underwent dehydration and blocking with 3% BSA prior to immunofluorescence staining. Subsequently, the sections were exposed to anti-CD68 antibody (1:200 dilution, GB113109, Servicebio) overnight at 4 °C, followed by incubation with Cy3-conjugated Goat Anti-Rabbit IgG (H+L) secondary antibody (1:300 dilution, GB21303, Servicebio) at room temperature for one hour in the absence of light. DAPI (1:1000, GDP1024, Servicebio) was utilized for nuclear counterstaining, with 10 min of incubation at room temperature in the dark. Images were scanned using an automatic digital slide scanner (3DHISTECH Pannoramic MIDI, Budapest, Hungary). Aipathwell software (version 1.0, Servicebio, Wuhan, China) was used to calculate the rate of positive cells.

#### 4.3.6. RNA Sequencing and Acquisition of Differentially Expressed Genes (DEGs)

Total RNA was extracted from the aorta using the TRIzol reagent kit (Invitrogen, Carlsbad, CA, USA). The quality of total RNA was evaluated using an Agilent 2100 Bioanalyzer (Agilent Technologies, Palo Alto, CA, USA) and further checked with RNase-free agarose gel electrophoresis. Following extraction, mRNA was enriched using Oligo (dT) beads and subsequently fragmented into shorter fragments through the use of a fragmentation buffer. The cleaved RNA fragments were reverse-transcribed into cDNA to create a final cDNA library using an NEBNext Ultra RNA Library Prep Kit for Illumina (NEB, New England Biolabs Ltd., Ipswich, MA, USA). The resulting cDNA library was sequenced using the Illumina Novaseq 6000 platform by Gene Denovo Biotechnology Co (Guangzhou, China). The FPKM (fragments per kilobase of exon model per million reads mapped) method was executed to represent gene expression levels. DESeq2 software (version 1.24.0) was used for differential expression analysis. Genes meeting the criteria of a false discovery rate (FDR) ≤ 0.05 and a log2 (Fold Change) ≥ 2 were identified as differentially expressed genes (DEGs).

#### 4.3.7. Identifying the Active Targets of FDT against Atherosclerosis

The DEG-potential target network was generated using Cytoscape 3.7.2 software (Cytoscape Consortium, San Diego, CA, USA) based on network pharmacology combined with transcriptome analysis. This intersection was selected as the active target of FDT against atherosclerosis.

#### 4.3.8. Molecular Docking

Molecular docking was conducted to investigate the binding interactions of the absorbed components with SELP and CCL2. First, based on network pharmacology, four absorbed constituents, salvianolic acids A, B, and C and rosmarinic acid, were selected as ligands for SELP and CCL2. The chemical structures of the four absorbed constituents were obtained from PubChem. In addition, the three-dimensional (3D) structures of SELP and CCL2 were acquired from the PDB Protein database. Molecular docking was conducted using CB-Dock2 [61]. The interaction mode of the docking results was visualized through the utilization of PyMol software (PyMOL Molecular Graphics System, Version 2.0 Schrödinger, LLC).

#### 4.3.9. Statistical Analysis

Data are presented as the mean ± standard error of mean (SEM), and a one-way analysis of variance (ANOVA) was conducted using GraphPad Prism software (version 8.0). A *p* value less than 0.05 was deemed to be statistically significant.

## 5. Conclusions

Through a combination of UFLC-TOF-MS, network pharmacology, and experimental validation, we found that FDT attenuated atherosclerosis and exerted anti-inflammatory effects by suppressing SELP and CCL2 gene expression, contributing to monocyte adhesion and migration. The inhibition of monocyte recruitment by FDT is a new perspective to elucidate the anti-atherosclerotic mechanism of FDT, which has not been adopted in previous studies on FDT. Our results may help elucidate the therapeutic mechanism of FDT against CVDs and provide potential therapeutic targets for CVDs.

## Figures and Tables

**Table 1:** Enriched GO biological process/KEGG signaling pathway of the intersection targets.

Modules	Type	GO Biological Process/KEGG Signaling Pathway	*p* Value
Immune/inflammation	GO	Positive regulation of cell migration	3.86 × 10^−11^
	GO	Positive regulation of blood vessel endothelial cell migration	5.53 × 10^ −4 ^
	GO	Positive regulation of mononuclear cell migration	1.51 × 10^ −4 ^
	GO	Leukocyte tethering or rolling	3.44 × 10^ −5 ^
	GO	Leukocyte cell–cell adhesion	3.22 × 10^ −6 ^
	GO	Positive regulation of inflammatory response	6.44 × 10^ −8 ^
	KEGG	Lipid and atherosclerosis	2.82 × 10^ −11 ^
	KEGG	IL-17 signaling pathway	5.45 × 10^ −6 ^
	KEGG	TNF signaling pathway	1.14 × 10^ −7 ^
Oxidative stress	GO	Response to oxidative stress	6.15 × 10^ −4 ^
	KEGG	Fluid shear stress and atherosclerosis	1.76 × 10^ −11 ^
Cell apoptosis	GO	Positive regulation of apoptotic process	5.40 × 10^ −10 ^
	GO	Negative regulation of apoptotic process	4.55 × 10^ −5 ^
Energy metabolism	GO	Response to hypoxia	1.77 × 10^ −14 ^
	GO	Cellular response to hypoxia	6.45 × 10^−6^
	KEGG	HIF-1 signaling pathway	1.45 × 10^−5^

**Table 2:** The Vina score of absorbed constituents and target proteins.

Target	PDB ID	Absorbed Constituents	Vina Score
SELP	1G1Q	Salvianolic acid A	−8.1
		Salvianolic acid B	−8.2
		Salvianolic acid C	−8.7
CCL2	1DOM	Rosmarinic acid	−6.5

## Data Availability

Data is contained within the article or Supplementary Material.

## Supplementary Materials

The following supporting information can be downloaded at https://www.mdpi.com/article/10.3390/ph17050643/s1, Table S1: Ingredients of Fufang Danshen Tablet (FDT); Table S2: Identification of the chemical constituents in Fufang Danshen Tablet (FDT); Table S3: Absorbed constituents in rat serum after the oral administration of Fufang Danshen Tablet (FDT); Table S4: The targets of absorbed constituents; Table S5: The targets of atherosclerosis; Table S6: The intersecting targets of FDT and atherosclerosis; Table S7: GO enrichment of intersecting targets; Table S8: KEGG enrichment of intersecting targets; Table S9: The differentially expressed genes (DEGs) between Model and FDT group in transcriptomics; Table S10: Chemicals and reagents used in UFLC-Q-TOF-MS/MS; Figure S1: Diagram of absorbed constituent–target–disease network.
