# Supplementary material for: Exploring the Mechanism of Fufang Danshen Tablet against Atherosclerosis by Network Pharmacology and Experimental Validation
Source: Pharmaceuticals (Basel). 2024 May 16;17(5):643. doi: 10.3390/ph17050643 (PMC11124970; doi:10.3390/ph17050643)
Supplement: Supplementary file 1 [file pharmaceuticals-17-00643-s001.zip › Table S1.pdf]

Table S1. Ingredients of Fufang Danshen Tablet (FDT)

Chinese name	Herb	Latin name	Medicinal part
Dan-Shen	*Salvia miltiorrhiza* Bunge	Salviae Miltiorrhizar Radix et Rhizoma	Dried roots and rhizome
San-Qi	*Panax notoginseng* (Burkill) F. H. Chen ex C. H.	Notoginseng Radix et Rhizoma	Dried roots and rhizome
Bing-Pian	/	Borneolum syntheticum	/
