# Supplementary material for: Exploring the Mechanism of Fufang Danshen Tablet against Atherosclerosis by Network Pharmacology and Experimental Validation
Source: Pharmaceuticals (Basel). 2024 May 16;17(5):643. doi: 10.3390/ph17050643 (PMC11124970; doi:10.3390/ph17050643)
Supplement: Supplementary file 1 [file pharmaceuticals-17-00643-s001.zip › Table S10.pdf]

Table S10. Chemicals and reagents used in UFLC-Q-TOF-MS/MS

Chemicals and reagents	Supplier
Notoginsenoside R_1	National Institute for Control of Biological and Pharmaceutical Products of China (Beijing, China)
Ginsenoside Rb_1	
Ginsenoside Rg_1	
Ginsenoside Re	
Ginsenoside Rg_3	
Salvianolic acid B	
Salvianolic acid C	
Protocatechuic aldehyde	
Cryptotanshinone	
Tanshinone II_A	
Danshensu sodium salt	
Caffeic acid	
Rosmarinic acid	
Protocatechuic acid	
Protopanaxatriol	Yuanye Bio-Technology Co., Ltd (Shang hai, China)
Lithospermic acid	
Dihydrotanshinone I	
Salvianolic acid A	
Salvianolic acid D	
Ginsenoside Rk_2	
Ginsenoside Rk_3	
Ginsenoside Rd	
Ginsenoside Rg_2	
Tanshinone I	
[2’,3’,5’,6’-D_4]-naringin	Artis-chem Co. Ltd (Shanghai, China)
HPLC-grade acetonitrile	Thermo Fisher (Fair Lawn, NJ, USA)
HPLC-grade methanol	
HPLC-grade formic acid	Macklin (Shanghai, China)
Fufang Danshen Tablet	Hutchison Whampoa Baiyunshan Chinese Medicine Co., Ltd (Guangzhou, China)
Atorvastatin calcium tablets	Pfizer Pharmaceuticals LLC (Vega Baja, USA)
