# Supplementary material for: Exploring the Mechanism of Fufang Danshen Tablet against Atherosclerosis by Network Pharmacology and Experimental Validation
Source: Pharmaceuticals (Basel). 2024 May 16;17(5):643. doi: 10.3390/ph17050643 (PMC11124970; doi:10.3390/ph17050643)
Supplement: Supplementary file 1 [file pharmaceuticals-17-00643-s001.zip › Table S2.pdf]

Table S2. Identification of the chemical constituents in Fufang Danshen Tablet (FDT)

N.	Identification	T_R (min)	Formula	$[M+H]^+$ (error, ppm)	$[M-H]^-$ (error, ppm)	Fragment ions in positive (+) ion mode	Fragment ions in negative (–) ion mode	Source
**1**	Stachyose	2.11	$C_{24}H_{42}O_{21}$	667.2295 (0.5)	665.2167 (3.2)	487.1631$[M+H-Glc-H_2O]^+$<br>325.1135$[M+H-2Glc-H_2O]^+$	485.1509$[M-H-Glc-H_2O]^-$	DS
**2**	Sucrose	2.18	$C_{12}H_{22}O_{11}$	343.1234 (-0.3)	341.1088 (-0.5)	307.1228$[M+H-2H_2O]^+$<br>163.0587$[M+H-Glc-H_2O]^+$<br>145.0485$[M+H-Glc-2H_2O]^+$<br>127.0380$[M+H-Glc-3H_2O]^+$	179.0556$[M-H-Glc]^-$<br>119.0343$[M-H-Glc-COOH-CH_3]^-$	DS
**3**	Raffinose	2.24	$C_{18}H_{32}O_{16}$	505.1758 (-0.9)	503.1616 (-0.2)	325.1137$[M+H-Glc-H_2O]^+$<br>163.0598$[M+H-2Glc-H_2O]^+$	ND	DS
**4**	Dencichin*	2.30	$C_5H_8N_2O_5$	177.0507 (0.8)	ND	160.0250$[M+H-NH_3]^+$<br>116.0345$[M+H-CO_2-NH_3]^+$<br>88.0380$[M+H-CO_2-NH_3-CO]^+$	ND	SQ
**5**	Adenosine	2.32	$C_{10}H_{13}N_5O_4$	268.1037 (-1.0)	ND	136.0615$[M+H-C_5H_8O_4]^+$	ND	SQ
**6**	Pyroglutamate	2.83	$C_5H_7NO_3$	130.0494 (-2.6)	128.0353 (-0.4)	84.0439$[M+H-H_2O-CO]^+$	82.0265$[M-H-H_2O-CO]^-$	DS
**7**	Succinic acid*	3.05	$C_4H_6O_4$	ND	117.0193 (-0.4)	ND	99.0077$[M-H-H_2O]^-$<br>73.0290$[M-H-CO_2]^-$	DS
**8**	Danshensu*	4.17	$C_9H_{10}O_5$	ND	197.0464 (1.9)	ND	179.0348$[M-H-H_2O]^-$<br>135.0447$[M-H-CO_2-H_2O]^-$<br>123.0446$[M-H-2CO-H_2O]^-$	DS
**9**	Protocatechuic acid*	4.84	$C_7H_6O_4$	ND	153.0193 (-0.4)	ND	109.0281$[M-H-CO_2]^-$<br>108.0195$[M-H-COOH]^-$	DS
**10**	Pseudoginsenoside Rt_5*	4.94	$C_{36}H_{62}O_{10}$	655.4408 (-0.6)	ND	637.4310$[M+H-H_2O]^+$<br>457.3622$[M+H-2H_2O-Glc]^+$<br>439.3566$[M+H-3H_2O-Glc]^+$<br>143.1053$[Glc-H_2O]^+$	ND	SQ
**11**	Ginsenoside F_1	5.61	$C_{36}H_{62}O_9$	639.4459 (-0.5)	ND	621.4290$[M+H-H_2O]^+$<br>603.4302$[M+H-2H_2O]^+$<br>441.3713$[M+H-2H_2O-Glc]^+$<br>423.3638$[M+H-3H_2O-Glc]^+$	ND	SQ
**12**	Unknow	5.63	$C_{22}H_{32}O_{13}$	ND	503.1771 (0.1)	ND	341.1249$[M-H-Glc]^-$	SQ
**13**	Protocatechualdehyde*	5.68	$C_7H_6O_3$	139.0385 (-3.5)	137.0244 (0.1)	93.0325$[M+H-H_2O-CO]^+$<br>65.0386$[M+H-CHO-COOH]^+$	136.0159$[M-H-H]^-$<br>108.0204$[M-H-CHO]^-$<br>92.0259$[M-H-COOH]^-$	DS

**14**	Caffeic acid*	6.01	$C_9H_8O_4$	ND	179.0351<br>(0.6)	ND	135.0445[M-H-CO$_2$]$^-$<br>134.0358[M-H-COOH]$^-$	DS
**15**	Quercetin	6.03	$C_{15}H_{10}O_7$	303.0501<br>(0.4)	ND	257.0448[M+H-H$_2$O-CO]$^+$<br>229.0480[M+H-H$_2$O-2CO]$^+$<br>183.0456[M+H-C$_4$H$_8$O$_4$]$^+$	ND	SQ
**16**	Quercetin 3-sambubioside	6.04	$C_{26}H_{28}O_{16}$	597.1449<br>(-0.3)	595.1322<br>(2.9)	465.1012[M+H-Ara]$^+$<br>303.0500[M+H-Ara-Glc]$^+$	301.0361[M-H-Glc-Ara]$^-$	SQ
**17**	Notoginsenoside C	6.19	$C_{54}H_{92}O_{25}$	ND	1139.5919<br>(4.9)	ND	1093.5882[M-H-H$_2$O-CO]$^-$	SQ
**18**	Yunnaneic acid D	6.48	$C_{27}H_{24}O_{12}$	ND	539.1206<br>(2.1)	ND	521.1001[M-H-H$_2$O]$^-$<br>359.0785[M-H-C$_9$H$_8$O$_4$]$^-$<br>341.0680[M-H-DSS]$^-$<br>197.0457[DSS-H]$^-$<br>179.0329[C$_9$H$_8$O$_4$-H]$^-$	DS
**19**	Salviaflaside	6.53	$C_{24}H_{26}O_{13}$	523.1436<br>(-2.0)	521.1312<br>(-0.3)	361.0946[M+H-Glc]$^+$<br>325.0949[M+H-Glc-2H$_2$O]$^+$<br>163.0387[Glc+H]$^+$	359.0775[M-H-Glc]$^-$<br>323.0782[M-H-DSS]$^-$<br>197.0437[DSS-H]$^-$<br>179.0334[CA-H]$^-$	DS
**20**	Yunnaneic acid F	6.55	$C_{29}H_{26}O_{14}$	ND	597.1264<br>(1.7)	ND	553.1315[M-H-CO$_2$]$^-$<br>417.0781[M-H-C$_9$H$_8$O$_4$]$^-$<br>197.0431[DSS-H]$^-$<br>179.0431[C$_9$H$_8$O$_4$-H]$^-$	DS
**21**	Notoginsenoside T$_5$	6.94	$C_{41}H_{68}O_{12}$	753.4778<br>(-0.7)	ND	441.3729[M+H-Xyl-Glc-H$_2$O]$^+$<br>423.3624[M+H-Xyl-Glc-2H$_2$O]$^+$	ND	SQ
**22**	Notoginsenoside R$_1$*	6.96	$C_{47}H_{80}O_{18}$	933.5420<br>(0.3)	931.5311<br>(4.2)	753.4961[M+H-Glc-H$_2$O]$^+$<br>621.4225[M+H-Glc-H$_2$O-Xyl]$^+$<br>441.3763[M+H-2Glc-Xyl-2H$_2$O]$^+$<br>423.3599[M+H-2Glc-Xyl-3H$_2$O]$^+$	799.4881[M-H-Xyl]$^-$	SQ
**23**	Salvianolic acid D*	7.17	$C_{20}H_{18}O_{10}$	ND	417.0831<br>(1.0)	ND	197.0451[DSS-H]$^-$<br>179.0338[C$_9$H$_8$O$_4$-H]$^-$<br>174.0291[M-H-DSS-COOH]$^-$	DS
**24**	Ginsenoside Re*	7.39	$C_{48}H_{82}O_{18}$	947.5573<br>(-0.2)	ND	767.4891[M+H-Glc-H$_2$O]$^+$<br>441.3710[M+H-2Glc-Rha-2H$_2$O]$^+$<br>423.3576[M+H-2Glc-Rha-3H$_2$O]$^+$<br>405.3512[M+H-2Glc-Rha-4H$_2$O]$^+$	ND	SQ
**25**	Ginsenoside Rg$_1$*	7.54	$C_{42}H_{72}O_{14}$	801.4990<br>(-0.6)	ND	621.4339[M+H-Glc-H$_2$O]$^+$<br>441.3739[M+H-2Glc-2H$_2$O]$^+$<br>423.3614[M+H-2Glc-3H$_2$O]$^+$	ND	SQ
**26**	Rosmarinic acid*	8.13	$C_{18}H_{16}O_8$	361.0916<br>(-0.4)	359.0781<br>(2.0)	163.0384[M+H-DSS]$^+$<br>145.0275[M+H-DSS-H$_2$O]$^+$	197.0453[DSS-H]$^-$<br>179.0344[CA-H]$^-$<br>161.0239[M-H-DSS]$^-$	DS

**27**	Salvianolic acid A*	8.66	$C_{26}H_{22}O_{10}$	ND	493.1156<br>(2.0)	ND	313.0720[M-H-CA]$^-$<br>295.0621[M-H-DSS]$^-$<br>197.0446[DSS-H]$^-$	DS
**28**	Lithospermic acid*	8.66	$C_{27}H_{22}O_{12}$	539.1179<br>(-0.9)	537.1050<br>(2.2)	521.1080[M+H-H_2O]$^+$<br>323.0545[M+H-H_2O-DSS]$^+$	313.0728[M-H-CA-CO_2]$^-$<br>295.0617[M-H-DSS-CO_2]$^-$<br>197.0455[DSS-H]$^-$	DS
**29**	Salvianolic acid B*	9.66	$C_{36}H_{30}O_{16}$	719.1602<br>(-0.6)	717.1491<br>(4.2)	521.1070[M+H-DSS]$^+$<br>493.1097[M+H-DSS-CO]$^+$<br>323.0551[M+H-DSS-$C_9H_8O_4$-H_2O]$^+$	519.0946[M-H-DSS]$^-$<br>339.0516[M-H-DSS-$C_9H_8O_4$]$^-$<br>321.0416[M-H-DSS-$C_9H_8O_4$-H_2O]$^-$	DS
**30**	Salvianolic acid A isomer	11.38	$C_{26}H_{22}O_{10}$	ND	493.1148<br>(1.7)	ND	313.0741[M-H-CA]$^-$<br>295.0611[M-H-DSS]$^-$<br>179.0345[CA-H]$^-$	DS
**31**	Dimethyl Lithospermate	13.98	$C_{29}H_{26}O_{12}$	567.1495<br>(-0.4)	565.1370<br>(2.1)	369.0980[M+H-DSS]$^+$<br>295.0604[M+H-DSS-2CO-H_2O]$^+$	519.0971[M-H-H_2O-CO]$^-$<br>367.0843[M-H-DSS]$^-$<br>339.0514[M-H-DSS-CO]$^-$<br>321.0406[M-H-DSS-CO-H_2O]$^-$	DS
**32**	Ethyl lithospermate	15.61	$C_{29}H_{26}O_{12}$	567.1500<br>(0.5)	565.1370<br>(2.1)	521.1096[M+H-CO-H_2O]$^+$<br>493.1017[M+H-2CO-H_2O]$^+$<br>323.0580[M+H-CO-H_2O-DSS]$^+$	367.0820[M-H-DSS]$^-$<br>321.0410[M-H-DSS-CO-H_2O]$^-$	DS
**33**	Notoginsenoside Fa*	18.67	$C_{59}H_{100}O_{27}$	ND	1239.6455<br>(4.4)	ND	1239.6504<br>1107.6153[M-H-Xyl]$^-$	SQ
**34**	Notoginsenoside R_2/isomer	18.79	$C_{41}H_{70}O_{13}$	ND	769.4778<br>(4.4)	ND	637.4372[M-H-Xyl]$^-$<br>475.3788[M-H-Xyl-Glc]$^-$	SQ
**35**	Ginsenoside Rb_1*	18.88	$C_{54}H_{92}O_{23}$	1109.6101<br>(-0.1)	1107.6025<br>(3.4)	767.4943[M+H-2Glc-H_2O]$^+$<br>605.4400[M+H-3Glc-H_2O]$^+$<br>425.3778[M+H-4Glc-2H_2O]$^+$	1107.6039	SQ
**36**	Ginsenoside Rh_3	18.89	$C_{36}H_{60}O_7$	605.4408<br>(-0.7)	ND	587.4431[M+H-H_2O]$^+$<br>425.3914[M+H-H_2O-Glc]$^+$<br>407.3692[M+H-2H_2O-Glc]$^+$	ND	SQ
**37**	Ginsenoside Rg_2*	18.89	$C_{42}H_{72}O_{13}$	785.5049<br>(0.5)	783.4927<br>(1.1)	605.4372[M+H-ORha-H_2O]$^+$<br>425.3756[M+H-ORha-Glc-2H_2O]$^+$	637.4336[M-H-Rha]$^-$	SQ
**38**	Malonylginsenoside Rb_1	18.99	$C_{57}H_{94}O_{26}$	1195.6106<br>(0)	1193.6006<br>(3.6)	1015.4980[M+H-H_2O-Glc]$^+$<br>835.4798[M+H-2Glc-2H_2O]$^+$<br>673.4200[M+H-3Glc-2H_2O]$^+$	1149.6158[M-H-CO_2]$^-$<br>1107.6050[M-H-$C_3H_2O_3$]$^-$	SQ
**39**	Notoginsenoside A	19.07	$C_{54}H_{92}O_{24}$	ND	1123.5947<br>(1.8)	ND	1077.5916[M-H-H_2O-CO]$^-$	SQ
**40**	Ginsenoside Rk_3*	19.15	$C_{36}H_{60}O_8$	621.4355<br>(-0.9)	ND	603.4289[M+H-H_2O]$^+$<br>441.3725[M+H-Glc-H_2O]$^+$<br>423.3631[M+H-Glc-2H_2O]$^+$<br>405.3492[M+H-Glc-3H_2O]$^+$	ND	SQ

**41**	Salvianolic acid F	19.15	$C_{17}H_{14}O_6$	ND	313.0717<br>(-0.1)	ND	161.0226[CA-H-H_2O]$^-$<br>151.0417[CA-H-CO]$^-$<br>133.0289[M-H-CA]$^-$	DS
**42**	Ginsenoside Rd*	19.25	$C_{48}H_{82}O_{18}$	947.5569<br>(-0.6)	945.5462<br>(3.5)	767.4859 [M+H-Glc-H_2O]$^+$<br>605.4373 [M+H-2Glc-H_2O]$^+$<br>425.3763 [M+H-3Glc-2H_2O]$^+$	945.5477	SQ
**43**	Ginsenoside Rk$_2^*$	19.27	$C_{36}H_{60}O_7$	605.4411<br>(-0.2)	ND	587.4269[M+H-H_2O]$^+$<br>551.4097[M+H-3H_2O]$^+$<br>425.3812[M+H-H_2O-Glc]$^+$<br>407.3680[M+H-2H_2O-Glc]$^+$	ND	SQ
**44**	Acetyl-ginsenoside F$_1$	19.35	$C_{38}H_{64}O_{10}$	ND	679.4454<br>(4.0)	ND	619.4284[M-H-HOAc]$^-$	SQ
**45**	Hydroxycryptotanshinone/<br>isomer	19.40	$C_{19}H_{20}O_4$	313.1435<br>(0.2)	ND	295.1332[M+H-H_2O]$^+$<br>277.1239[M+H-2H_2O]$^+$<br>267.1372[M+H-H_2O-CO]$^+$<br>255.0994[M+H-2CH_3-CO]$^+$	ND	DS
**46**	Salvianolic acid C*	19.42	$C_{26}H_{20}O_{10}$	ND	491.0988<br>(0.9)	ND	447.1073[M-H-CO_2]$^-$<br>293.0480[M-H-DSS]$^-$<br>197.0433[DSS-H]$^-$<br>179.0342[$C_9H_8O_4$-H]$^-$	DS
**47**	20(S)-sanchirhinosides A$_2$	20.09	$C_{43}H_{72}O_{14}$	ND	811.4894<br>(3.8)	ND	765.4835[M-H-CO-H_2O]$^-$<br>619.4240[M-H-2CH_3-Glc]$^-$	SQ
**48**	Tanshinol B	20.22	$C_{18}H_{16}O_4$	297.1122<br>(0.1)	ND	279.1010[M+H-H_2O]$^+$<br>261.0905[M+H-2H_2O]$^+$<br>233.0959[M+H-2H_2O-CO]$^+$	ND	DS
**49**	Ginsenoside Rg$_3$	20.45	$C_{42}H_{72}O_{13}$	785.5048<br>(0.3)	783.4929<br>(2.2)	749.5063[M+H-2H_2O]$^+$<br>443.3958[M+H-2Glc-H_2O]$^+$<br>425.3758[M+H-2Glc-2H_2O]$^+$	621.4244[M-H-Glc]$^-$	SQ
**50**	3β-<br>Hydroxymethylenetanshinquinone	20.48	$C_{18}H_{14}O_4$	295.0965<br>(0.1)	293.0817<br>(-1)	249.0914[M+H-CO-H_2O]$^+$<br>221.0956[M+H-H_2O-2CO]$^+$	265.0877[M-H-CO]$^-$<br>250.0647[M-H-CO-CH_3]$^-$	DS
**51**	Tanshinone II$_B$	20.59	$C_{19}H_{18}O_4$	311.1279<br>(0.4)	ND	267.1382[M+H-CO_2]$^+$<br>252.1153[M+H-CO_2-CH_3]$^+$	ND	DS
**52**	Salvianonol	20.62	$C_{18}H_{20}O_4$	301.1433<br>(-0.4)	ND	283.1334[M+H-H_2O]$^+$<br>265.1238[M+H-2H_2O]$^+$<br>241.1232[M+H-COOH-CH_3]$^+$	ND	DS
**53**	Hydroxytanshinone	20.80	$C_{19}H_{18}O_4$	311.1281<br>(0.9)	ND	293.1183[M+H-H_2O]$^+$<br>275.1074[M+H-2H_2O]$^+$	ND	DS
**54**	Nortanshinone	20.81	$C_{17}H_{12}O_4$	281.0807<br>(-0.3)	ND	235.0757[M+H-H_2O-CO]$^+$<br>207.0808[M+H-H_2O-2CO]$^+$	ND	DS
**55**	Didehydrotanshinone II$_A$	20.82	$C_{19}H_{16}O_3$	293.1172<br>(-0.1)	ND	247.0749[M+H-H_2O-CO]$^+$<br>219.0803[M+H-H_2O-2CO]$^+$	ND	DS

**56**	Panaxydol	21.17	$C_{17}H_{24}O_2$	261.1847<br>(-0.8)	ND	168.9769[M+H-C_6H_4O]$^+$<br>141.1260[M+H-C_6H_4O-CO]$^+$	ND	SQ
**57**	Danshenxinkun A	21.30	$C_{18}H_{16}O_4$	297.1123<br>(0.4)	295.0981<br>(1.5)	261.0911[M+H-2H_2O]$^+$<br>233.0967[M+H-2H_2O-CO]$^+$	265.0856[M-H-2CH_3]$^-$<br>237.0923[M-H-2CH_3-CO]$^-$	
**58**	Isocryptotanshinone	21.52	$C_{19}H_{20}O_3$	297.1486<br>(0.3)	ND	253.1585[M+H-CH_3-CHO]$^+$<br>238.1347[M+H-CHO-2CH_3]$^+$	ND	DS
**59**	Neocryptotanshinone isomer	21.53	$C_{19}H_{22}O_4$	315.1591<br>(-0.1)	313.1450<br>(1.4)	297.1493[M+H-H_2O]$^+$<br>253.1592[M+H-H_2O-CHO-CH_3]$^+$<br>238.1350[M+H-H_2O-CHO-2CH_3]$^+$	269.1549[M-H-CHO-CH_3]$^-$<br>213.1280[M-H-CHO-CH_3-2CO]$^-$	DS
**60**	Przewaquinone B	21.57	$C_{18}H_{18}O_4$	299.1279<br>(0.3)	297.1135<br>(0.9)	281.1193[M+H-H_2O]$^+$<br>263.1066[M+H-2H_2O]$^+$<br>235.1086[M+H-2H_2O-CO]$^+$	253.1228[M-H-CHO-CH_3]$^-$<br>209.1338[M-H-2CHO-2CH_3]$^-$	DS
**61**	Dehydromiltirone/ Sibiriquinone A	21.90	$C_{19}H_{20}O_2$	281.1536<br>(0.2)	ND	263.1409[M+H-H_2O]$^+$<br>252.1154[M+H-CHO]$^+$<br>238.0980[M+H-CH_3-CO]$^+$	ND	DS
**62**	Hydroxycryptotanshinone/ isomer	22.00	$C_{19}H_{20}O_4$	313.1433<br>(-0.5)	ND	295.1345[M+H-H_2O]$^+$<br>277.1248[M+H-2H_2O]$^+$<br>269.1536[M+H-CHO-CH_3]$^+$<br>251.1433[M+H-H_2O-CHO-CH_3]$^+$	ND	DS
**63**	Ginsenoside Rg_5*	22.21	$C_{42}H_{70}O_{12}$	767.4934<br>(-0.7)	765.4825<br>(4.0)	605.4399[M+H-Glc]$^+$<br>425.3786[M+H-2Glc-H_2O]$^+$<br>407.3653[M+H-2Glc-2H_2O]$^+$	719.2866[M-H-CO-H_2O]$^-$<br>603.4265[M-H-Glc]$^-$	SQ
**64**	Dihydrotanshinone I*	22.46	$C_{18}H_{14}O_3$	279.1013<br>(-0.9)	ND	233.0967[M+H-CO-H_2O]$^+$<br>205.1011[M+H-2CO-H_2O]$^+$<br>190.0780[M+H-2CO-H_2O-CH_3]$^+$	ND	DS
**65**	Horminone	22.66	$C_{20}H_{28}O_4$	333.2064<br>(1.0)	331.1918<br>(1.0)	315.1917[M+H-H_2O]$^+$<br>297.1904[M+H-2H_2O]$^+$	301.1810[M-H-2CH_3]$^-$	DS
**66**	Norsalvioxide	22.66	$C_{18}H_{24}O_2$	273.1847<br>(-0.7)	271.1707<br>(1.1)	255.1746[M+H-H_2O]$^+$<br>243.1738[M+H-2CH_3]$^+$<br>199.1101[M+H-3CH_3-CHO]$^+$	241.1595[M-H-2CH_3]$^-$<br>199.0769[M-H-CO-CHO-CH_3]$^-$	DS
**67**	Neocryptotanshinone	22.75	$C_{19}H_{22}O_4$	315.1590<br>(-0.2)	313.1449<br>(1.2)	297.1479[M+H-H_2O]$^+$<br>279.1374[M+H-2H_2O]$^+$<br>254.0931[M+H-H_2O-CO-CH_3]$^+$	283.1351[M-H-2CH_3]$^-$<br>267.1396[M-H-CO-H_2O]$^-$<br>255.1398[M-H-2CH_3-CO]$^-$	DS
**68**	Microstegiol	22.78	$C_{20}H_{26}O_2$	299.2003<br>(-0.8)	297.1861<br>(0.5)	257.1543[M+H-C_3H_6]$^+$<br>229.1227[M+H-CO-C_3H_6]$^+$	282.1627[M-H-CH_3]$^-$<br>254.1321[M-H-CO-CH_3]$^-$	DS
**69**	Isotanshinone II_A	23.34	$C_{19}H_{18}O_3$	295.1329<br>(0)	ND	277.1215[M+H-H_2O]$^+$<br>225.1268[M+H-3CH_2-CO]$^+$	ND	DS
**70**	Epidanshenspiroketallactone	23.51	$C_{17}H_{16}O_3$	269.1171<br>(-0.4)	ND	251.1061[M+H-H_2O]$^+$<br>223.1118[M+H-H_2O-CO]$^+$<br>195.1153[M+H-H_2O-2CO]$^+$	ND	DS
**71**	Horminon isomer	24.07	$C_{20}H_{28}O_4$	333.2066	331.1923	315.1944[M+H-H_2O]$^+$	313.1817[M-H-H_2O]$^-$	DS

				(1.6)	(2.4)	287.2042[M+H-H_2O-CO]$^+$	298.1568[M-H-CH_3-H_2O]$^-$<br>270.1630[M-H-CH_3-H_2O-CO]$^-$	
**72**	Sugiol	24.35	$C_{20}H_{28}O_2$	301.2160<br>(-0.6)	299.2023<br>(2.3)	259.1689[M+H-C_3H_6]$^+$<br>213.1277[M+H-H_2O-CO-C_3H_6]$^+$	227.1084[M-H-C_3H_6-2CH_3]$^-$	DS
**73**	Cryptotanshinone*	24.43	$C_{19}H_{20}O_3$	297.1487<br>(0.5)	ND	254.0942[M+H-CO-CH_3]$^+$<br>251.1428[M+H-CO-H_2O]$^+$	ND	DS
**74**	Tanshinone I *	24.59	$C_{18}H_{12}O_3$	277.0858<br>(-0.5)	ND	249.0906[M+H-CO]$^+$<br>178.0776[M+H-3CO-CH_3]$^+$	ND	DS
**75**	Miltirone I	25.06	$C_{18}H_{16}O_2$	265.1221<br>(-0.8)	ND	223.0756[M+H-C_3H_6]$^+$<br>204.0929[M+H-CO-H_2O-CH_3]$^+$<br>195.0800[M+H-C_3H_6-CO]$^+$	ND	DS
**76**	Dihydrotanshinone I isomer	25.59	$C_{18}H_{14}O_3$	279.1015<br>(-0.4)	ND	261.0905[M+H-H_2O]$^+$<br>205.1012[M+H-2CO-H_2O]$^+$	ND	DS
**77**	Dehydromiltirone/ Sibiriquinone A	26.62	$C_{19}H_{20}O_2$	281.1535<br>(-0.2)	ND	221.0963[M+H-4CH_3]$^+$<br>193.1014[M+H-4CH_3-CO]$^+$	ND	DS
**78**	Tanshinone II_A*	27.24	$C_{19}H_{18}O_3$	295.1328<br>(-0.3)	ND	277.1220[M+H-H_2O]$^+$<br>252.0790[M+H-CO-CH_3]$^+$<br>249.1265[M+H-H_2O-CO]$^+$	ND	DS
**79**	Miltirone	28.17	$C_{19}H_{22}O_2$	283.1694<br>(0.4)	ND	223.1121[M+H-HOAc]$^+$<br>208.0889[M+H-CH_3- HOAc]$^+$	ND	DS
**80**	Salviolone	28.45	$C_{18}H_{20}O_2$	269.1535<br>(-0.4)	ND	254.1302[M+H-CH_3]$^+$<br>239.1059[M+H-2CH_3]$^+$	ND	DS
**81**	Oleanolic acid	31.92	$C_{30}H_{48}O_3$	457.3677<br>(0.2)	455.3534<br>(0.8)	439.3573[M+H-H_2O]$^+$<br>411.3653[M+H-H_2O-CO]$^+$	455.3528	DS
