# Supplementary material for: Exploring the Mechanism of Fufang Danshen Tablet against Atherosclerosis by Network Pharmacology and Experimental Validation
Source: Pharmaceuticals (Basel). 2024 May 16;17(5):643. doi: 10.3390/ph17050643 (PMC11124970; doi:10.3390/ph17050643)
Supplement: Supplementary file 1 [file pharmaceuticals-17-00643-s001.zip › Table S3.pdf]

Table S3 Absorbed constituents in rat serum after the oral administration of Fufang Danshen Tablet

No.	Identification	T_R (min)	Formula	$[M+H]^+$ (error, ppm)	$[M-H]^-$ (error, ppm)	Characteristic Fragment	
						POS	NEG
P1	Danshensu*	4.14	$C_9H_{10}O_5$	ND	197.0458 (0.3)	ND	179.0350$[M-H-H_2O]^-$<br>152.8940$[M-H-COOH]^-$<br>135.0441$[M-H-CO_2-H_2O]^-$
P2	Ginsenoside F_1	5.64	$C_{36}H_{62}O_9$	639.4442 (-3.9)	ND	621.4514$[M+H-H_2O]^+$<br>441.3689$[M+H-2H_2O-Glc]^+$<br>423.3499$[M+H-3H_2O-Glc]^+$	ND
P3	Salvianolic acid D*	7.15	$C_{20}H_{18}O_{10}$	ND	417.0823 (-1.1)	ND	197.0419$[DSS-H]^-$<br>179.0346$[C_9H_8O_4-H]^-$
P4	Ginsenoside Rg_1*	7.47	$C_{42}H_{72}O_{14}$	801.4996 (0.1)	ND	621.4332$[M+H-H_2O-Glc]^+$<br>441.3698$[M+H-2H_2O-2Glc]^+$<br>423.3593$[M+H-3H_2O-2Glc]^+$<br>405.3513$[M+H-4H_2O-2Glc]^+$	ND
P5	Rosmarinic acid*	8.14	$C_{18}H_{16}O_8$	ND	359.0775 (0.8)	ND	197.0439$[DSS-H]^-$<br>161.0253$[M-H-DSS]^-$
P6	Salvianolic acid A*	8.83	$C_{26}H_{22}O_{10}$	ND	493.1154 (2.7)	ND	313.0682$[M-H-CA]^-$<br>295.0601$[M-H-DSS^-$<br>197.0518$[DSS-H]^-$
P7	Lithospermic acid*	8.84	$C_{27}H_{22}O_{12}$	ND	537.1046 (1.3)	ND	493.1177$[M-H-CO_2]^-$<br>295.0615$[M-H-DSS-CO_2]^-$
P8	Salvianolic acid B*	11.02	$C_{36}H_{30}O_{16}$	ND	717.1493 (4.5)	ND	519.0924$[M-H-DSS]^-$<br>339.0507$[M-H-DSS-CA]^-$<br>321.0400$[M-H-2DSS]^-$

P9	Ginsenoside Rb_1*	18.91	$C_{54}H_{92}O_{23}$	1109.6155 (4.8)	1107.6043 (7.8)	767.4861[M+H-2Glc-H_2O]$^+$<br>325.1125[2Glc+H]$^+$	1107.6041[M-H]$^-$
P10	Ginsenoside Rd*	19.25	$C_{48}H_{82}O_{18}$	947.5579 (0.6)	945.5454 (2.7)	767.4939[M+H-Glc-H_2O]$^+$<br>605.4418[M+H-2Glc-H_2O]$^+$<br>425.3773[M+H-3Glc-2H_2O]$^+$	945.5396[M-H]$^-$<br>783.4673[M-H-Glc]$^-$
P11	Salvianolic acid C*	19.65	$C_{26}H_{20}O_{10}$	ND	491.0989 (1.1)	ND	293.0443[M-H-DSS]$^-$<br>197.0516[DSS-H]$^-$
P12	Przewaquinone B	19.85	$C_{18}H_{12}O_4$	293.0811 (0.8)	ND	249.0876[M+H-CO_2]$^+$<br>178.0767[M+H-2CO-CH_3-CO_2]$^+$	ND
P13	Tanshinol B	20.22	$C_{18}H_{16}O_4$	297.1124 (0.9)	ND	279.1039[M+H-H_2O]$^+$<br>261.0918[M+H-2H_2O]$^+$<br>233.0976[M+H-2H_2O-CO]$^+$	ND
P14	Tanshinone II_B	20.57	$C_{19}H_{18}O_4$	311.1278 (0)	ND	267.1373[M+H-CO_2]$^+$<br>252.1152[M+H-CO_2-CH_3]$^+$	ND
P15	Salvianonol	20.62	$C_{18}H_{20}O_4$	301.1427 (-0.1)	ND	283.1345[M+H-H_2O]$^+$<br>265.1228[M+H-2H_2O]$^+$<br>241.1209[M+H-COOH-CH_3]$^+$	ND
P16	Danshenxinkun A	21.31	$C_{18}H_{16}O_4$	297.1122 (0.1)	295.0979 (1.2)	261.0912[M+H-2H_2O]$^+$<br>233.0963[M+H-2H_2O-CO]$^+$	265.0863[M-H-2CH_3]$^-$<br>237.0917[M-H-2CH_3-CO]$^-$
P17	Dihydrotanshinone I*	22.43	$C_{18}H_{14}O_3$	279.1014 (-0.5)	ND	261.0903[M+H-H_2O]$^+$<br>205.1004[M+H-2CO-H_2O]$^+$	ND
P18	Neocryptotanshinone	22.76	$C_{19}H_{22}O_4$	315.1594 (0.9)	313.1444 (-0.5)	297.1488[M+H-H_2O]$^+$<br>279.1372[M+H-2H_2O]$^+$	283.135 [M-H-2CH_3]$^-$<br>255.1404[M-H-2CH_3-CO]$^-$
P19	Epidanshenspiroketallactone	23.50	$C_{17}H_{16}O_3$	269.1172 (0)	ND	251.1089[M+H-H_2O]$^+$<br>223.1100[M+H-H_2O-CO]$^+$<br>177.0068[M+H-2H_2O-2CO]$^+$	ND

P20	Cryptotanshinone*	24.42	$C_{19}H_{20}O_3$	297.1488 (0.9)	ND	282.1314[M+H-CH_3]$^+$ 251.1449[M+H-CO-H_2O]$^+$	ND
P21	Tanshinone I*	24.52	$C_{18}H_{12}O_3$	277.0858 (-0.3)	ND	249.0914[M+H-CO]$^+$ 178.0779[M+H-3CO-CH_3]$^+$	ND
P22	Tanshinone II_A*	27.24	$C_{19}H_{18}O_3$	295.1326 (-1)	ND	277.1217[M+H-H_2O]$^+$ 252.0777[M+H-CO-CH_3]$^+$ 249.1264[M+H-H_2O-CO]$^+$	ND

Note: *Positively identified via comparison with authentic standards. ND, Not detected; DSS, $C_9H_{10}O_5$ (Danshensu); CA, $C_9H_8O_4$ (Caffeic acid); Glc, glucose; GlcUA, glucuronyl moitety.
