# Supplementary figures and images for: Exploring the Mechanism of Fufang Danshen Tablet against Atherosclerosis by Network Pharmacology and Experimental Validation
Source: Pharmaceuticals (Basel). 2024 May 16;17(5):643. doi: 10.3390/ph17050643 (PMC11124970; doi:10.3390/ph17050643)

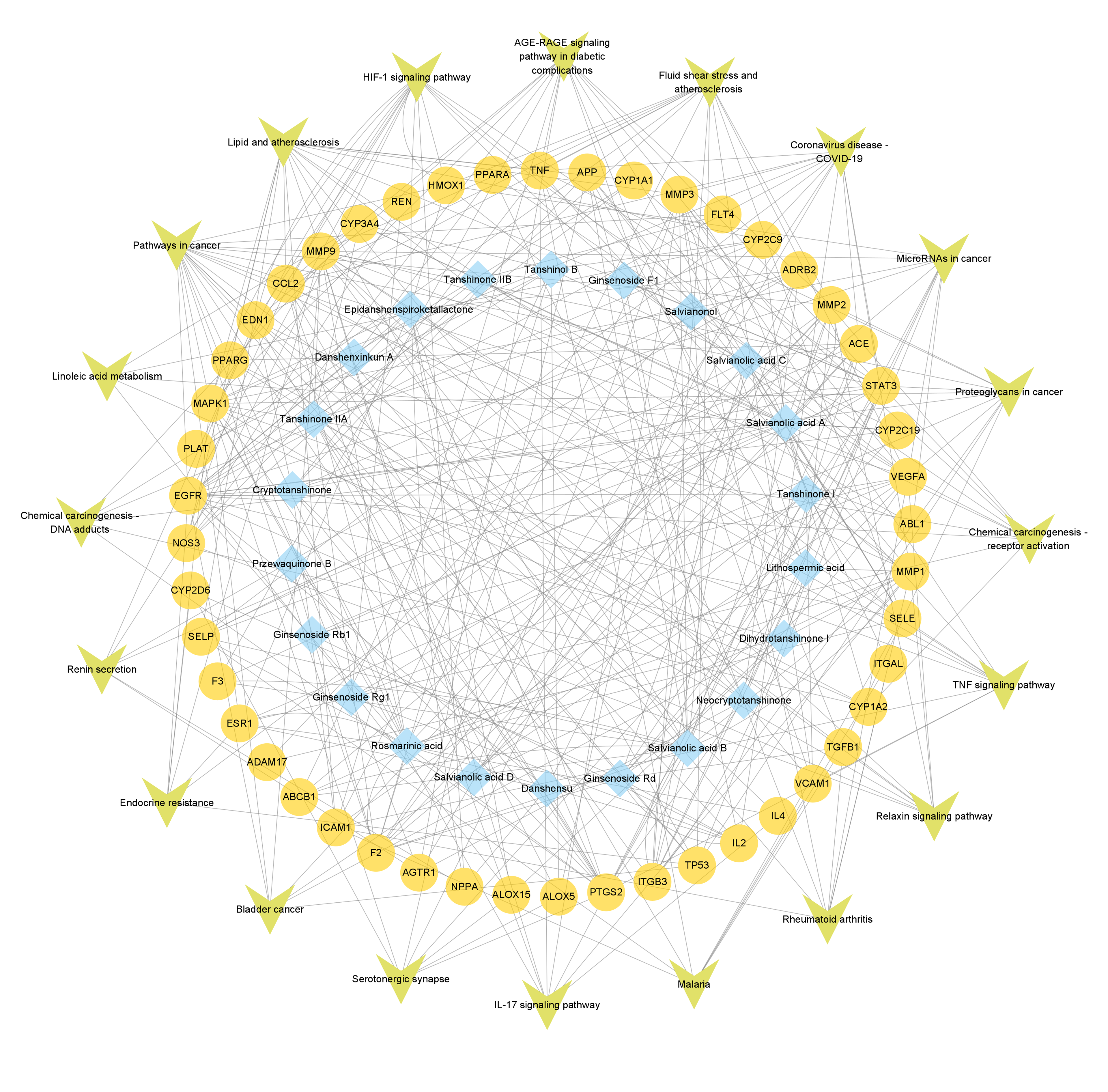

Supplement: Supplementary file 1 [file pharmaceuticals-17-00643-s001.zip › Figure S1.tif]
